# β-Catenin Controls the Electrophysiologic Properties of Skeletal Muscle Cells by Regulating the α2 Isoform of Na^+^/K^+^-ATPase

**DOI:** 10.3389/fnins.2019.00831

**Published:** 2019-08-07

**Authors:** Congying Zhao, Yonglin Yu, Yi Zhang, Jue Shen, Lihua Jiang, Guoxia Sheng, Weiqin Zhang, Lu Xu, Kewen Jiang, Shanshan Mao, Peifang Jiang, Feng Gao

**Affiliations:** ^1^Department of Neurology, Children’s Hospital, School of Medicine, Zhejiang University, Hangzhou, China; ^2^Department of Rehabilitation, Children’s Hospital, School of Medicine, Zhejiang University, Hangzhou, China; ^3^Department of Biobank, Children’s Hospital, School of Medicine, Zhejiang University, Hangzhou, China; ^4^Department of Neurobiology, Key Laboratory of Medical Neurobiology of Ministry of Health of China, Zhejiang Province Key Laboratory of Neurobiology, School of Medicine, Zhejiang University, Hangzhou, China; ^5^Scientific Research Office, Children’s Hospital, School of Medicine, Zhejiang University, Hangzhou, China

**Keywords:** electrophysiologic properties, neuromuscular junction, skeletal muscle, Na^+^/K^+^-ATPase, β-catenin

## Abstract

β-Catenin is a key component of the canonical Wnt signaling pathway. It has been shown to have an important role in formation of the neuromuscular junction. Our previous studies showed that in the absence of β-catenin, the resting membrane potential (RMP) is depolarized in muscle cells and expression of the α2 subunit of sodium/potassium adenosine triphosphatase (α2NKA) is reduced. To understand the underlying mechanisms, we investigated the electrophysiologic properties of a primary cell line derived from mouse myoblasts (C2C12 cells) that were transfected with small-interfering RNAs and over-expressed plasmids targeting β-catenin. We found that the RMP was depolarized in β-catenin knocked-down C2C12 cells and was unchanged in β-catenin over-expressed muscle cells. An action potential (AP) was not released by knockdown or over-expression of β-catenin. α2NKA expression was reduced by β-catenin knockdown, and increased by β-catenin over-expression. We showed that β-catenin could interact physically with α2NKA (but not with α1NKA) in muscle cells. NKA activity and α2NKA content in the cell membranes of skeletal muscle cells were modulated positively by β-catenin. These results suggested that β-catenin (at least in part) regulates the RMP and AP in muscle cells, and does so by regulating α2NKA.

## Introduction

β-Catenin is a crucial downstream component of the Wnt signaling pathway. β-Catenin translocates to the nucleus and subsequently regulates target–gene transcription (Molenaar et al., 1996; Hecht et al., 2000). β-Catenin also binds to the cytoplasmic domain of α-catenin and cadherins, where it participates in cell adherence and organization of the actin cytoskeleton (Nelson and Nusse, 2004). β-Catenin not only promotes cell proliferation and tissue expansion, it also affects the fate and final differentiation of cells after cell division. β-Catenin is associated with disorders caused by abnormal development and some tumors (Chilosi et al., 2003; Varallo et al., 2003; Jamieson et al., 2004; Batlle et al., 2005). The function of β-catenin is extensive, but it is associated with several diseases. However, up until now, the underlying mechanisms have not been clear.

In the central nervous system (CNS), β-catenin participates in synaptic assembly: neural development, axonal growth, orientation, and synaptogenesis (Salinas, 2003; Yoshikawa et al., 2003; Zou, 2004). Studies have suggested that Wnt/β-catenin signaling is involved in Alzheimer’s disease (Caricasole et al., 2003; Zhao et al., 2005), schizophrenia (Singh et al., 2010), and emotional disorders (Lovestone et al., 2007).

In the peripheral nervous system, the neuromuscular junction (NMJ), which is the synapse between a motor neuron and a muscle fiber, plays an important part in muscle contraction. Appropriate interactions between motor neurons and muscle cells are required for information transmission at the NMJ (Sanes and Lichtman, 2001; Wu et al., 2010).

Agrin is released by motor neurons and binds to low-density lipoprotein receptor-related protein (LRP)-4 and activates muscle-specific kinase (MuSK). Agrin, LRP4, and MuSK are needed for NMJ formation (DeChiara et al., 1996; Gautam et al., 1996; Zong et al., 2012). In contrast, muscle cells produce retrograde signals for presynaptic differentiation (Lyuksyutova et al., 2003). It has been suggested that β-catenin in muscle might play a key part in the formation and development of the NMJ (Zhang et al., 2007; Li et al., 2008; Wang et al., 2008; Wu et al., 2015).

Research has revealed that most mutant mice deficient in β-catenin (and specifically deficient in β-catenin in skeletal muscles) die a few hours after birth (Li et al., 2008; Zhao et al., 2014). Previously, we showed that β-catenin is crucial for maintenance of the resting membrane potential (RMP) of skeletal muscle cells, and that its lack of expression can induce marked reductions in expression of the α2 subunit of sodium/potassium adenosine triphosphatase (α2NKA) (Zhao et al., 2014).

In the present study, we showed that β-catenin plays a part in the RMP and action potential (AP) of skeletal muscle cells at the NMJ. We recorded depolarization following knockdown of the β-catenin gene, but variance was not seen if β-catenin was over-expressed in skeletal muscle cells. The AP was inhibited by up-regulation and down-regulation of functional expression of β-catenin. Also, α2NKA expression was reduced by knocking down β-catenin and was increased by over-expressing β-catenin. We found that α2NKA and β-catenin displayed a “physical” interaction in muscle cells. Finally, NKA activity and α2NKA content in the cell membrane were found to be modulated positively by β-catenin.

We were able to suggest a mechanism underlying the electrophysiologic changes caused by β-catenin expression in muscle cells. We also provided additional evidence that supports a key role for β-catenin at the NMJ. Our data could be used to advance research on the intricate role of β-catenin in neurologic disorders.

## Materials and Methods

### Reagents and Antibodies

Antibodies targeted against β-catenin and α2NKA were obtained from Abcam (AB32572; used at 1:6000 dilution for immunoblotting and 1:200 for staining; AB2871, used at 1:500 for immunoblotting and 1:50 for staining; Cambridge, United Kingdom). Antibodies against K^+^-voltage-gated channel subfamily C member 4 (Kv3.4; DF10312; 1:1000) and glyceraldehyde-3-phosphate dehydrogenase (GAPDH; AF7021; 1:1000) were purchased from Affinity (Cincinnati, OH, United States). Antibodies against α1NKA (GTX22867; 1:600) were obtained from GeneTex (Irvine, CA, United States). Horseradish peroxidase (HRP)-conjugated goat anti-mouse immunoglobulin (Ig)G (BA1051; 1:50,000), HRP-conjugated goat anti-rabbit IgG (BA1054; 1:50,000), Cy3-conjugaed goat anti-rabbit IgG (BA1032; 1:2000), and fluorescein isothiocyanate (FITC)-conjugated goat anti-mouse IgG (BA1101; 1:2000) were purchased from Wuhan Boster (Wuhan City, China). A Na^+^/K^+^-ATP Enzyme Test kit (BC0060) was obtained from Solarbio Life Sciences (Beijing, China). A Cell Surface Protein Isolation kit (89881) was purchased from Pierce (Rockford, IL, United States).

### Constructs

Chemically modified small interfering (si)RNA oligonucleotides were obtained from Shanghai GenePharma (Shanghai, China). The 5′-termini of all siRNAs were labeled with a fluorescent conjugated dye (carboxyfluorescein). The sequences of β-catenin siRNAs (sense and antisense, respectively) were: 5′-CACCU CCCAA GUCCU UUAUT T-3′ and 5′-AUAAA GGACU UGGGA GGUGT T-3′ for siRNA-459; 5′-CCAGG UGGUA GUUAA UAAAT T-3′ and 5′-UUUAU UAACU ACCAC CUGGT T-3′ for siRNA-777; 5′-GGGUU CCGAU GAUAU AAAUT T-3′ and 5′-AUUUA UAUCA UCGGA ACCCT T-3′ for siRNA-1512; 5′-UUCUC CGAAC GUGUC ACGUT T-3′ and 5′-ACGUG ACACG UUCGG AGAAT T-3′ for the negative control (carboxyfluorescein).

The cDNA of β-catenin was obtained by reverse transcription-polymerase chain reaction (RT-PCR) using the primer sequences (forward and reverse, respectively) 5′-GGACT CAGAT CTCGA GATGG CTACT CAAGC TGACC T-3′ and 5′-GTCGA CTGCA GAATT CTTAC AGGTC AGTAT CAAAC CAG-3′. This was followed by sub-cloning into the pIRES2-ZsGreen1 plasmid (Supplementary Figure 1). The overexpressed plasmid we constructed was called pIRES2-ZsGreen1-β-catenin.

### Culture and Transfection of Cells

C2C12 myoblasts were obtained from American Type Culture Collection (Manassas, VA, United States). They were cultured in high-glucose Dulbecco’s modified Eagle’s medium (DMEM; 11965118; Invitrogen, Carlsbad, CA, United States) supplemented with 100 U/mL penicillin–streptomycin, 10% fetal bovine serum (16000-044; Invitrogen, Carlsbad, CA, United States), and 1 mM GlutaMAX (Thermo Fisher, Boston, MA, United States) in 60-mm dishes. Fusion of myoblasts into myotubes was induced by culture in differentiation medium (high-glucose DMEM supplemented with 2% horse serum). Myoblasts at 70–80% confluence were transfected with Lipofectamine 2000 (11668019; Invitrogen, Carlsbad, CA, United States) according to manufacturer instructions, and then switched to differentiation medium 24-h later.

### Electrophysiology Experiment

Fully differentiated myotubes transfected with siRNAs and pIRES2-ZsGreen1-β-catenin were visualized with an inverted microscope (AE31E; Motic, Beijing, China). They were recorded using whole-cell recording techniques employing an amplifier (EPC10), a Patchmaster v2 × 73 analog-to-digital converter, and IGOR 6.0.1.0 (all from HEKA Elektronik, Berlin, Germany). Recording pipettes were pulled to a tip resistance of 3–5 MΩ when filled with an internal solution containing (in mM): 140 potassium gluconate, 5 NaCl, 0.1 CaCl_2_, 1 EGTA, 10 HEPES, 1 MgCl_2_, and 2 Mg-ATP (pH 7.2 with KOH, and 295 mOsm). Cells were perfused throughout recordings with an external solution containing (in mM): 140 NaCl, 3.5 KCl, 1 MgCl_2_, 2 CaCl_2_, 10 D-glucose, 10 HEPES, and 1.25 NaH_2_PO_4_ (pH 7.4 with NaOH and 300 mOsm). Only cells with elongated (rather than flat) morphology were selected. Slightly positive pressure was applied while the pipette was advanced into the bath with the microelectrode manipulator. Once the glass pipette made contact with the cell surface, positive pressure was removed. Then, negative pressure was used to form a pipette-cell seal at >1 GΩ, and gentle suction was applied to rupture the patch membrane. The slow capacitance was compensated, and the membrane capacitance and series resistance were recorded. Seal formation and whole-cell recording were carried out in “current clamp” mode. The RMP was recorded at zero current immediately after breaking the seal between the membrane and the pipette. Cells with a series resistance <20 MΩ and changes <20% throughout recording were used for analyses. Current signals were Bessel-filtered at 5 kHz and sampled at 10 kHz.

Independent APs were evoked by intracellular injection of depolarizing current in a series of 600–1100 pA (at increments of 10–100 pA) rectangular pulses with 10 mS at 20 Hz (Li et al., 2015). All experiments were conducted at room temperature.

### Immunoblotting

C2C12 cells were collected and lysed with lysate buffer supplemented with a “protease inhibitor cocktail” [50 mM Tris–Cl (pH 7.4), 1 mM EDTA (pH 8.0), 250 mM NaCl, and 1% Triton-X] (Roche, Basel, Switzerland). The concentration of protein lysates was measured using the Bradford method (5000006; Bio-Rad Laboratories, Hercules, CA, United States). Subsequently, 10 μg of cell lysate with 5 μg of sample buffer [15 g of sodium dodecyl sulfate (SDS), 15.6 mL of 2 M Tris (pH 6.8), 57.5 g of glycerol, 16.6 mL of β-mercaptoethanol] was added to a 10% polyacrylamide gel. Samples were separated by 10% sodium dodecyl sulfate–polyacrylamide gel electrophoresis (SDS–PAGE) and transferred to polyvinylidene fluoride (PVDF) membranes (162-0177; Bio-Rad Laboratories, Hercules, CA, United States). After sealing with 4% milk containing 0.1% Tween, the PVDF membranes were incubated overnight with primary antibodies against β-catenin, α2NKA, KV3.4, α1NKA, and GAPDH, respectively, at 4°C. They were then washed three times with phosphate-buffered saline (PBS) containing 0.1% Tween. Next, 4% skimmed milk containing 0.1% Tween and HRP-conjugated antibody were added and incubated for 2 h at room temperature. After an electrochemiluminescence developer (170-5060; Bio-Rad Laboratories, Hercules, CA, United States) had been added to PVDF membranes, photographs were obtained using the Gel Doc^TM^ imaging system (Bio-Rad Laboratories, Hercules, CA, United States).

### Quantitative RT-PCR (qRT-PCR)

mRNA samples from C2C12 cells were prepared using an RNeasy mini-kit (74106; Qiagen, Stanford, VA, United States) and digested using an RNase-Free DNase kit (79254; Qiagen, Stanford, VA, United States). mRNA samples were reverse-transcribed into cDNA using a high-capacity cDNA Reverse Transcription kit (4368813) according to manufacturer (Applied Biosystems, Foster City, CA, United States) instructions. qRT-PCR was done with a SYBR^®^ Premix Ex Taq^TM^ II kit (DRR041A; TaKaRa Biotechnology, Shiga, Japan) in a real-time PCR instrument (QuantStudio 6; Applied Biosystems, Foster City, CA, United States) with pre-denaturation at 50°C for 10 min, denaturation at 95°C for 30 s, and annealing at 60°C for 30 s for a total of 40 cycles. The primer sequences (forward and reverse, respectively) were: 5′-CTTAC GGCTA CAGAG AGGGG-3′ and 5′-GCAGA GGGAA GCCGT AGTAT-3′ for α1NKA; 5′-ATCAA TGCAG AGGAG GTGGT-3′ and 5′-TGAAC TCAGG AGAAC GGGTC-3′ for α2NKA; 5′-AGACA GCTCG TTGTA CTGCT-3′ and 5′-GTGTC GTGAT GGCGT AGAAC-3′ for β-catenin; 5′-ATGGG TGTGA ACCAC GAGA-3′ and 5′-CAGGG ATGAT GTTCT GGGCA-3′ for GAPDH.

### Immunohistochemistry and Co-immunoprecipitation

C2C12 cells were fixed by immersion in 4% paraformaldehyde in PBS for 30 min at 4°C. After addition of Triton X-100 to a final concentration of 0.1%, sections were mounted on gelatin-coated glass slides. Non-specific binding sites were blocked by preincubation for 1 h at room temperature in bovine serum albumin in PBS (PBS/BSA), followed by incubation with the primary antibody in PBS/BSA overnight at 4°C. Bound antibody was visualized by labeling with a FITC-conjugated anti-rabbit antibody or Cy3-conjugated anti-mouse antibody. Secondary antibodies were used at 1:2000 dilution. Fluorescence images were captured using a confocal laser scanning microscope and software (C2; Nikon, Tokyo, Japan).

C2C12 cells were resuspended in immunoprecipitation buffer supplemented with protease inhibitors and phosphatase inhibitors for 10–15 min on ice, and centrifugated at 10,000 × *g* for 10 min at 4°C. The supernatant was precleared with 30 μL of protein A + G agarose beads for 2 h at 4°C to eliminate non-specific binding and reduce the background reading. Samples were incubated with 3 μg of β-catenin antibody at 4°C overnight. Immune complexes were harvested with another 30 μL of protein A + G agarose beads for 3–6 h at a 4°C. The beads were resuspended in 30 μL of 2× SDS–PAGE buffer and washed three times with PBS. α2NKA and α1NKA were immunoprecipitated under identical conditions using monoclonal antibodies. After heating for 5 min at 100°C in 2× sample buffer, immunoprecipitated proteins were subjected to SDS–PAGE and immunoblotting as described above.

### Assays to Measure Enzyme Activity

Cell suspensions of myoblasts at 80–90% confluence were thawed on ice and homogenized for 10 min at1000 × *g*. Then, the homogenate was centrifuged at 1000 × *g* for 10 min at 4°C to obtain the precipitate, which was then resuspended in 1 mL of homogenizing buffer [0.1 mol/L imidazole-HCl, 0.3 mol/L sucrose, and 1 g/L of sodium deoxycholate (without EDTA)] for 10 min at 37°C. The homogenized sample was centrifuged for 10 min at 4000 × *g* and 4°C. The supernatant obtained interfered with subsequent phosphate analyses, so it was mixed with a phosphorus reagent for 10 min in a 40°C water bath, and then cooled to room temperature. Absorbance was determined at 660 nM using an automatic microplate spectrophotometer (Multiskan MK3, Thermo Scientific).

Assays for Na^+^/K^+^-ATPase activity were determined as described by Ching et al. (2015) using the following formula:

ATPase activity (U/mg protein) = C standard × (A estimated – A control)/(A standard – A blank) × V total/(protein concentration of the sample × V sample)/T = 7.5 × (A estimated – A control)/(A standard – A blank)/protein concentration of the sample.

A, enzymatic activity (the amount of inorganic phosphorus produced by enzymatic decomposition of ATP per milligram of tissue protein and per hour as a unit of enzymatic activity); C, concentration, V, volume, T, the reaction time.

### Biotinylation on the Surface of Muscle Cells

Cells were starved with serum-free DMEM overnight, washed with ice-cold PBS, and incubated with PBS containing 0.5 mg/mL non-permeable EZ-link^TM^ Sulfo-NHS-SS-Biotin (pH 8.0; Thermo Scientific) on ice for 30 min. (EZ-link Sulfo-NHS-SS-Biotin is a membrane-impermeable reagent which forms a stable covalent linkage with an extended spacer arm to reduce the steric hindrance associated with avidin binding.) The liquid was suctioned. Cells were incubated with 4 mL of 100 mmol/L glycine at 4°C for 5 min. The step described above was repeated. Cells were washed and harvested by scraping in pre-cooled cell lysate buffer containing 1% Triton-X100 and protease inhibitors (150 mM NaCl, 5 mM EDTA, 50 mM Tris, pH 7.5). The supernatant was harvested and boiled in 4× buffer solution for 10 min. For pull down of biotinylated proteins, 500 μL of the protein extract was incubated with 30 μL of high-capacity streptavidin agarose beads (Neutravid Agarose; 29202; Pierce, Rockford, IL, United States) overnight at 4°C. Beads were washed, eluted by incubation in Laemmli buffer containing dithiothreitol at 100°C for 10 min, and analyzed by western immunoblotting. The lysate input and elutes were subjected to SDS–PAGE and analyzed by western immunoblotting.

### Statistical Analyses

Data are the mean ± SEM. Data were calculated using one-way analysis of variance (ANOVA) with Tukey’s honestly significantly different test. Changes were considered significant if alpha values satisfied the limit of *P* < 0.05.

## Results

### Deletion (but Not Over-Expression of) β-Catenin Depolarized the RMP in Cultured Muscle Cells

Our previous study showed that HSA-β-cat^–/–^ pups died hours after birth having presented with global cyanosis, consistent with previous reports (Li et al., 2008; Zhao et al., 2014). We wished to investigate further the role of β-catenin in muscle cells. Hence, we knocked down and over-expressed β-catenin in C2C12 cells by generating siRNAs targeting β-catenin (labeled with a fluorescent dye to mark successful transfection) and sub-cloning β-catenin cDNA into the pIRES2-ZsGreen1 plasmid, respectively. A siRNA that does not target any known mammalian gene was synthesized as a negative siRNA control. A blank pIRES2-ZsGreen1 plasmid was synthesized as a negative over-expression control.

**FIGURE 1:**
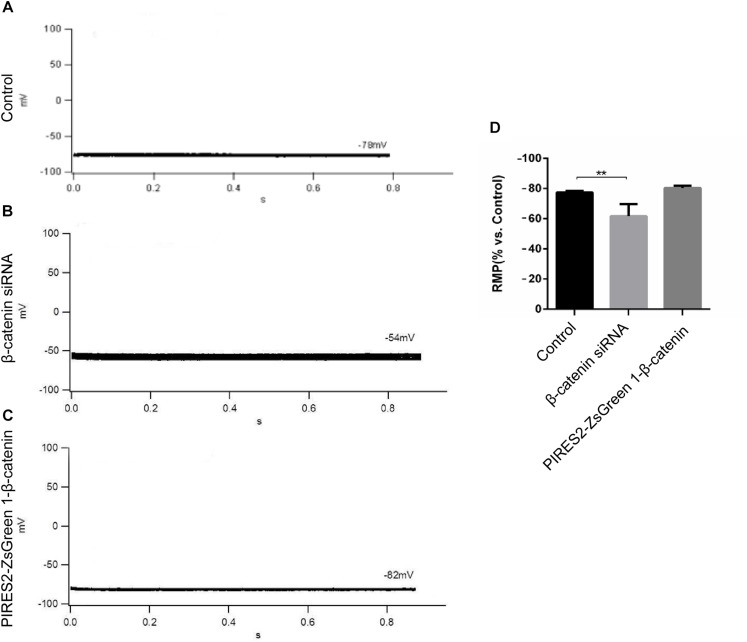
The RMP was depolarized in C2C12 muscle cells by knocking down β-catenin, but was not significantly changed by overexpressing β-catenin. **(A–C)** Whole-cell recordings of the RMP from C2C12 cells of control, β-catenin siRNA, and pIRES2-ZsGreen1-β-catenin, respectively. **(D)** Histograms show the absolute values of the RMP of β-catenin siRNA- and pIRES2-ZsGreen1-β-catenin-transfected C2C12 cells from more than three experiments with values from the control normalized as 1. *n* = 3 cells per group. Values are the mean ± SEM. ^∗∗^*P* < 0.01. Error bars represent SEM.

β-Catenin expression in cells transfected with β-catenin siRNAs and pIRES2-ZsGreen1-β-catenin plasmids was reduced and increased significantly, respectively (Supplementary Figures 2, 3, *t*-test, *P* = 0.0027 for β-catenin siRNA group vs. siRNA NC group, and *P* = 0.0014 for pIRES2-ZsGreen1-β-catenin group vs. pIRES2-ZsGreen1 group). β-Catenin siRNA459 and a number 1 bacterial solution of pIRES2-ZsGreen-β-catenin were selected for subsequent studies. To ascertain if the RMP of β-catenin siRNA- and pIRES2-ZsGreen1-β-catenin plasmid-transfected cells had changed, we undertook whole-cell patch-clamp recording. We found that, in control cells, the RMP was −76 ± 2.83 mV (Figure 1A), whereas the RMP of cells transfected with β-catenin siRNA459 and pIRES2-ZsGreen1-β-catenin was −63 ± 7.07 mV (one-way ANOVA, *P* = 0.00067) and −80.75 ± 1.50 mV, respectively (Figures 1B,C). These data showed notable depolarization in C2C12 cells in which the β-catenin gene was knocked down, and no significant change in cells in which β-catenin was over-expressed (Figure 1D).

### AP Was Inhibited by Up- and Down-Regulation of β-Catenin in C2C12 Cells

**FIGURE 2:**
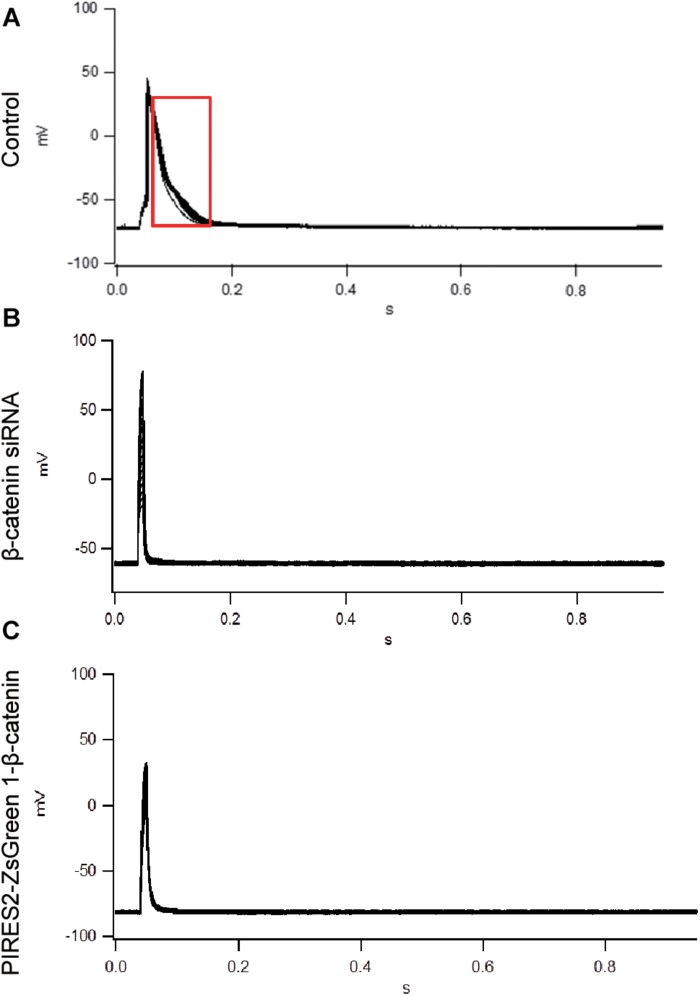
An AP could not be evoked by up- or down-regulation of β-catenin expression in C2C12 cells. **(A)** The AP of muscle cells evoked in control cells. **(B,C)** No AP was seen in β-catenin siRNA or pIRES2-ZsGreen1- β-catenin C2C12 cells. *n* = 3 cells per group.

To evoke an AP, current of duration 10 mS was delivered under a current clamp. To evoke an AP, electrical stimulation (600–1100 pA) was given (increased in increments of 10–100 pA). The AP of muscle cells was evoked in control cells, and the morphology and platform stage were normal (Figure 2A). However, in β-catenin knocked-down cells and over-expressed cells, an AP was not evoked in muscle cells (Figures 2B,C). Our results showed that the generation of AP required a proper β-catenin expression in the muscle cell membrane of NMJ.

### Expression and Transcription of α2NKA Were Associated Positively With β-Catenin in Muscle Cells

Our data showed that β-catenin knockdown depolarized skeletal muscle cells. Furthermore, the AP was not evoked in β-catenin knocked-down or in over-expressed C2C12 cells. Thus, we further explored the possible mechanisms underlying this phenomenon.

Sodium/potassium adenosine triphosphatase and Kv3.4 channels (Abbott et al., 2001) are crucial for maintaining the RMP in skeletal muscle cells. NKA is important for muscle excitability (Radzyukevich et al., 2013). Skeletal muscles in newborns mainly express α1NKA and α2NKA.

Moreover, β-catenin regulates the expression and transcription of target genes in the Wnt canonical pathway (Behrens et al., 1996; Clevers, 2006; Salinas and Zou, 2008; Budnik and Salinas, 2011). Thus, we ascertained if β-catenin functions in muscle cells by regulating expression of ion-channel proteins.

**FIGURE 3:**
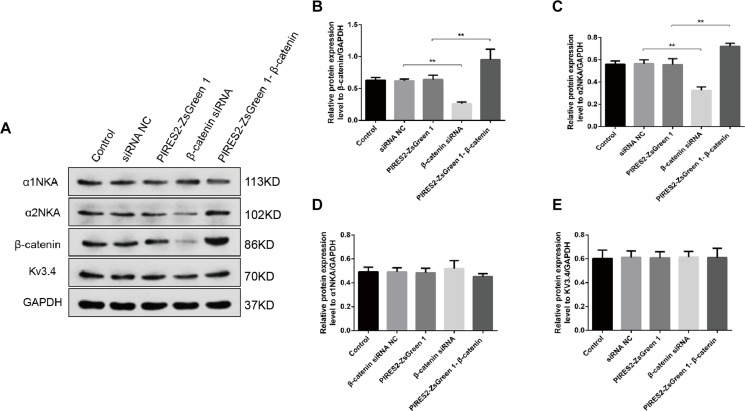
Protein expression of α2NKA, α1NKA, and Kv3.4 in each transfection group. **(A)** Representative expressions of α1NKA, α2NKA, β-catenin, Kv3.4 and GAPDH proteins in five independent groups respectively. Histograms show quantification of β-catenin **(B)**, α2NKA **(C)**, α1NKA **(D)**, and Kv3.4 **(E)** as protein levels relative to GAPDH from five independent experiments. The five groups are the control; siRNA normal control; pIRES2-ZsGreen1; β-catenin siRNA; pIRES2-ZsGreen1-β-catenin. *n* = 3 per group. ^*^P < 0.05, ^∗∗^*P* < 0.01. Error bars represent SEM.

**FIGURE 4:**
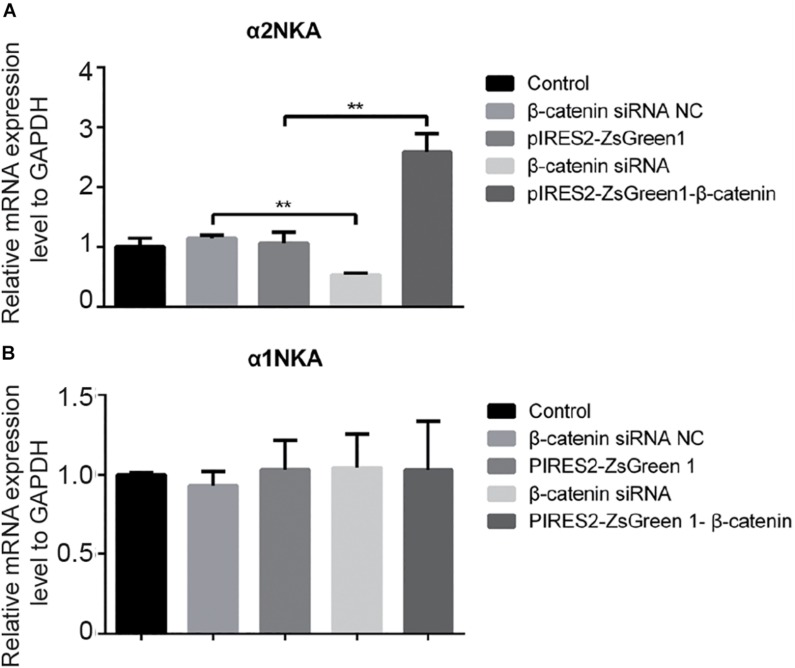
qPCR of α 2NKA mRNA **(A)** and α1NKA **(B)** mRNA in each transfection group. The five groups were: control; siRNA normal control; pIRES2-ZsGreen1; β-catenin siRNA; and pIRES2-ZsGreen1-β-catenin. mRNA expression was relative to that of GAPDH, and by changing the mean Δ*C*(*t*) value of each experimental group to Power 2^−ΔΔ^^*Ct*^. The threshold cycle (*Ct*) was obtained as the fractional cycle number at which the amount of amplified target reached a fix threshold. Data normalization was performed by subtracting *Ct* value of the GAPDH from that of the target gene. The ΔΔ*Ct* was calculated as the difference of the normalized *Ct* value (Δ*Ct*) of the experimental samples and control samples. ΔΔ*Ct* = Δ*Ct*_experiment_ – Δ*Ct*_control_. The control group was nominally considered as 1. *n* = 3 per group. *^∗∗^P* < 0.01, Error bars represent SEM.

We undertook immunoblotting and qRT-PCR in β-catenin knocked-down and over-expressed cells. We measured the protein expression of α1NKA and α2NKA in transfected C2C12 cells. Transfection with β-catenin siRNA caused a significant reduction of α2NKA expression (*P* < 0.0001 for β-catenin siRNA) (Figures 3A,C), and transfection with pIRES2-ZsGreen1-β-catenin caused a significant increase in α2NKA expression (*P* = 0.0002 for pIRES2-ZsGreen1-β-catenin) (Figures 3A,C). α2NKA expression was reduced to 57.5 ± 3.2% in β-catenin siRNA cells and increased to 129.2 ± 2.8% as compared with controls (Figure 3C). No significant difference was found in expression of α1NKA or Kv3.4 in any of the groups (Figures 3D,E). The decline in β-catenin expression reduced α2NKA transcription markedly (*P* = 0.0014 for β-catenin siRNA group vs. siRNA NC group, and *P* < 0.0001 for pIRES2-ZsGreen1-β-catenin group vs. pIRES2-ZsGreen1 group). By contrast, enhanced expression of β-catenin increased α2NKA transcription markedly (Figure 4).

These data suggested that β-catenin regulates RMP maintenance and partially triggers the AP by altering the transcription and expression of α2NKA in skeletal muscle cells.

### α2NKA and β-Catenin Interact Physically in Muscle Cells

**FIGURE 5:**
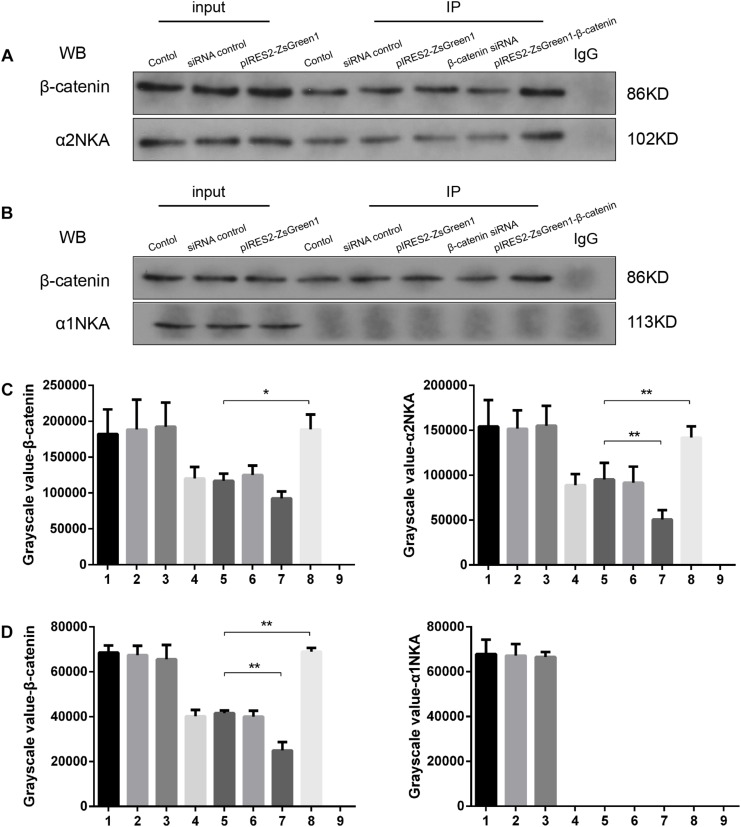
β-Catenin could pull down α2NKA protein **(A)** but not α1NKA protein **(B)** in C2C12 cells. 1. C2C12 input; 2. C2C12 siRNA control input; 3. C2C12 pIRES2-ZsGreen1 input; 4. C2C12 control immunoprecipitation; 5. C2C12 siRNA control immunoprecipitation; 6. C2C12 pIRES2-ZsGreen1 immunoprecipitation; 7. C2C12 β-catenin siRNA immunoprecipitation; 8. C2C12 pIRES2-ZsGreen1-β-catenin immunoprecipitation; 9. IgG control. Histograms show grayscale quantification of β-catenin (**C** left, **D** left), α2NKA (**C** right) and α1NKA (**D** right), from nine independent experiments. ^*^P < 0.05, *^∗∗^P* < 0.01, Error bars represent SEM.

We found that β-catenin transfection regulated α2NKA (but not α1NKA) expression positively, so we investigated if there was an interaction between α2NKA and β-catenin. Co-immunoprecipitation studies showed that β-catenin could pull down α2NKA protein but not α1NKA protein in C2C12 cells (Figure 5). Furthermore, the abundance of α2NKA protein changed according to β-catenin abundance (Figure 5A). When compared with the control group (Figure 5A, fourth group), the interaction between α2NKA and β-catenin did not induce obvious changes in β-catenin siRNA normal control cells (Figure 5A, fifth group) or in pIRES2-ZsGreen1 cells (Figure 5A, sixth group). Compared with siRNA normal control cells (Figure 5A, fifth group), the interaction between α2NKA and β-catenin was weakened in β-catenin siRNA cells (Figure 5A, seventh group). Compared with pIRES2-ZsGreen1 cells (Figure 5A, sixth group), the interaction between α2NKA and β-catenin was enhanced in pIRES2-ZsGreen1-β-catenin cells (Figure 5A, eighth group). In C2C12 control cells (Figure 5B, fourth group), β-catenin could not pull down α1NKA protein. Thus, in C2C12 siRNA control cells, C2C12 β-catenin over-expressed control cells, C2C12 β-catenin siRNA cells, and C2C12 β-catenin over-expressed cells, α1NKA protein could not be detected, and whether expression of β-catenin protein was decreased or increased was not known (Figure 5B). As expected, in cultured skeletal muscle cells, β-catenin co-localized with α2NKA, but it failed to do so with α1NKA. Thus, β-catenin could physically influence the abundance and activity of proteins (Figures 5C,D).

**FIGURE 6:**
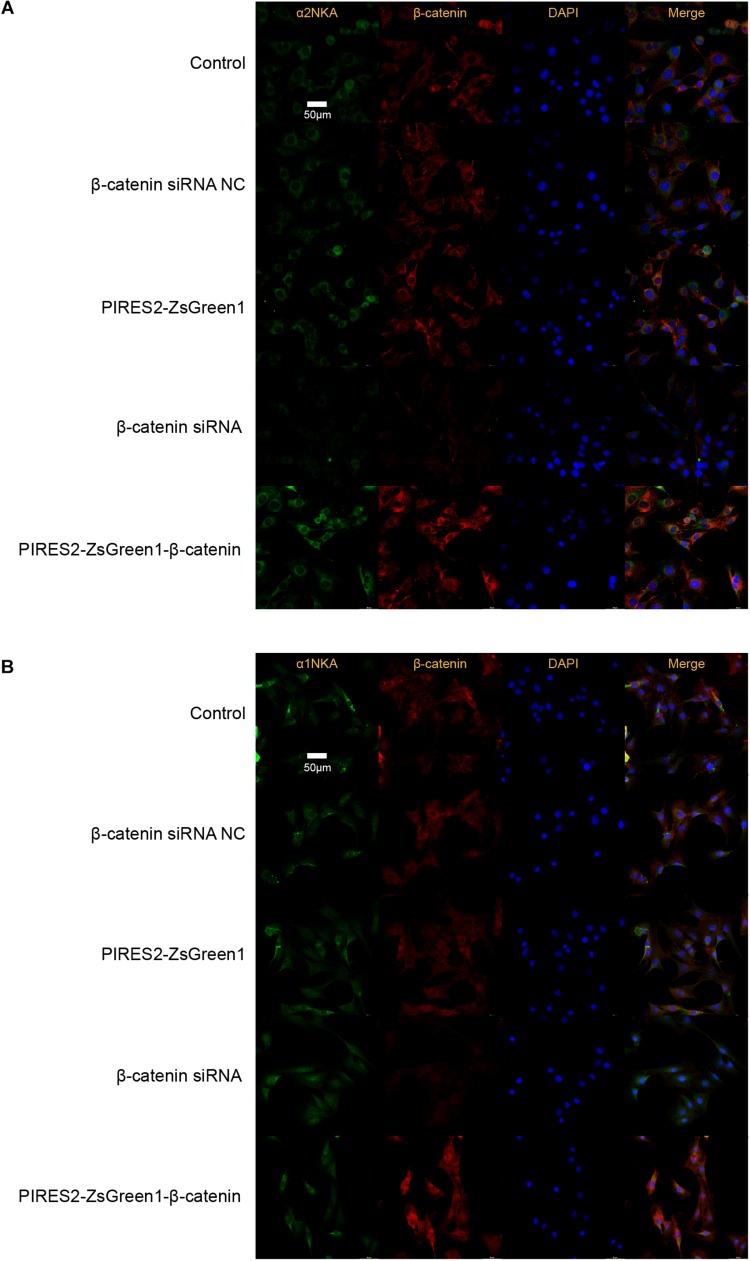
Co-localization of β-catenin and NKA in C2C12 cells, and expression of β-catenin, α2NKA, and α1NKA in C2C12 cells. **(A)** The immunofluorescence intensity of β-catenin and α2NKA in muscle cells from five groups is as described in Figure 3. **(B)** The immunofluorescence intensity of β-catenin and α1NKA in muscle cells from five groups is as described in Figure 3. Scale bar, 50 μm. *n* = 3 per group.

Confocal microscopy revealed that β-catenin protein co-localized with the proteins of α2NKA and α1NKA in C2C12 cells (Figure 6). In addition, α2NKA fluorescence was reduced in β-catenin knocked-down cells, and was increased in β-catenin over-expressed cells compared with control cells (Figure 6B). Otherwise, there was no change in the fluorescence of α1NKA protein compared with each C2C12 group (Figure 6A). These findings suggested that β-catenin could interact physically with α2NKA in muscle cells.

### NKA Activity Was Changed Throughout β-Catenin Expression in Muscle Cells

The results mentioned above showed that β-catenin regulates the electrophysiologic properties of muscle cells and α2NKA expression. In addition, NKA activity has an important role in maintenance of the RMP in skeletal muscles. We determined whether deletion and increases in β-catenin expression affected NKA activity.

**FIGURE 7:**
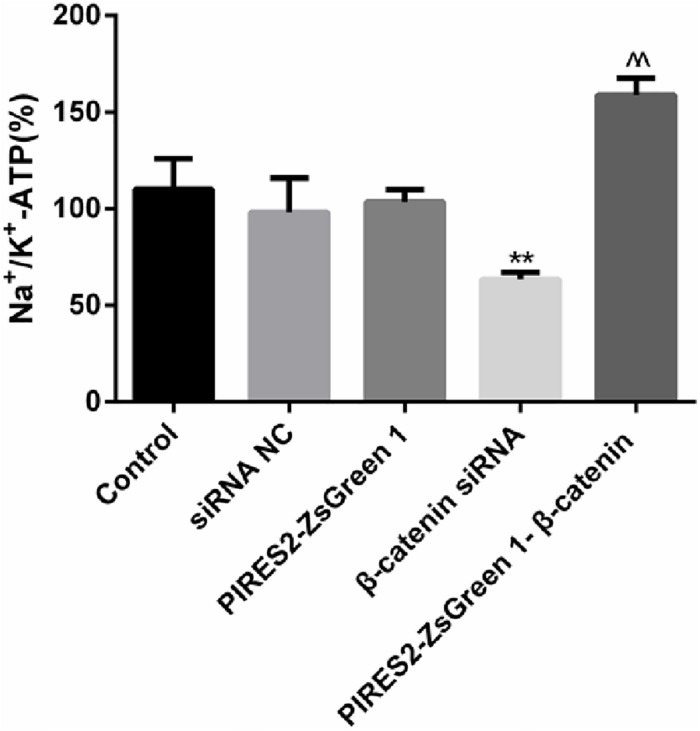
NKA activity changed throughout β-catenin expression in C2C12 cells. The experiment was designed according to the five groups described above. Relative activity from five independent experiments. ^∗∗^*P* < 0.01 vs. siRNA normal control group; ^^*P* < 0.01 vs. PIRES2-ZsGreen 1 group. Error bars represent SEM.

The percentage activity of NKA was not significantly different among the siRNA control group, PIRES2-ZsGreen1 group, or control group. When compared with siRNA control cells, the percentage activity of NKA was reduced significantly in the β-catenin knockdown group (Figure 7, *P* = 0.0046). Compared with the PIRES2-ZsGreen1 group, the percentage activity of NKA was increased significantly in the β-catenin over-expressed group (Figure 7, *P* = 0.0002).

Our results showed that interference with β-catenin inhibited NKA activity, whereas β-catenin over-expression promoted NKA activity.

### α2NKA Content in the Cell Membrane Was Regulated Positively by β-Catenin in Muscle Cells

Sodium/potassium adenosine triphosphatase is an integral protein in cell membranes. We measured the content of α2NKA and α1NKA in cell membranes by cell-surface biotinylation.

**FIGURE 8:**
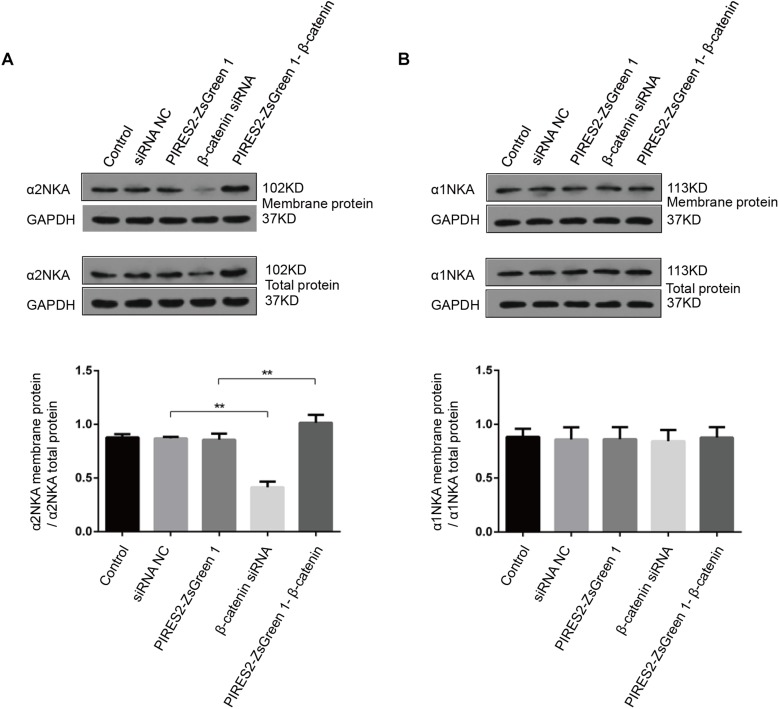
α2NKA content in the cell membrane was regulated by β-catenin expression in C2C12 cells. **(A)** α2NKA content in the cell membrane and total α2NKA protein in cells from five groups as described above (upper two lines). Histograms show quantification of α2NKA/total α2NKA protein in cell membranes from five independent experiments (bottom line). **(B)** Content of α1NKA in the cell membrane and total α1NKA protein in cells from five groups as described above (upper two lines). Histograms show quantification of membrane α1NKA/total α1NKA protein in the cell membrane from five independent experiments (bottom line). *n* = 3 per group. ^∗∗^*P* < 0.01 error bars represent SEM.

Compared with the siRNA control group, the content of total α2NKA, α2NKA in the cell membrane, and α2NKA/total α2NKA protein in the cell membrane was reduced significantly in cells treated with β-catenin siRNA (Figure 8A, *P* < 0.0001). However, the content of total α2NKA, α2NKA in the cell membrane, and α2NKA/total α2NKA protein in the cell membrane was not significantly different between the two groups (Figure 8B). In addition, when compared with the pIRES2-ZsGreen 1 control plasmid-transfected group, the content of total α2NKA, α2NKA in the cell membrane, and α2NKA/total α2NKA protein in the cell membrane was increased significantly in β-catenin over-expressed cells (Figure 8A, *P* = 0.0032). The content of total α2NKA, α2NKA in the cell membrane, and α2NKA/total α2NKA protein in the cell membrane was not significantly different when comparing the two groups (Figure 8B).

These data further demonstrated an interaction between β-catenin and α2NKA, but not with α1NKA. Over-expression of β-catenin could improve the expression and content of α2NKA in cell membranes. Furthermore, interference by β-catenin could reduce the expression and content of membrane-expressed α2NKA.

## Discussion

The present study demonstrated that β-catenin is involved in regulation of the RMP in skeletal muscle cells (Zhao et al., 2014). We revealed a novel mechanism underlying the early postnatal death of mice deficient in β-catenin in skeletal muscle. We also found that evoking the AP in skeletal muscle cells was affected by the deletion and over-expression of β-catenin.

We demonstrated that the protein expression and mRNA transcription of α2NKA were reduced if β-catenin in muscle cells was knocked down, and were increased if β-catenin in muscle cells was over-expressed. Furthermore, our results suggested that β-catenin and α2NKA interacted physically with muscle cells. Finally, we found that the activity of NKA and α2NKA content in the cell membrane was modulated positively by β-catenin.

These data suggested a possible mechanism underlying the changes in the RMP and AP concordant with changes in β-catenin in muscle cells, and provided additional evidence supporting a role for β-catenin at the NMJ.

### The Important Role of β-Catenin in Muscle Cells at NMJs

Increasing evidence suggests that Wnt/β-catenin signaling plays an important part in the development and maturation of the CNS (Wodarz and Nusse, 1998; Ciani and Salinas, 2005). Agrin-induced clustering of acetylcholine (ACh) receptors is inhibited by up- and down-regulation of presynaptic β-catenin at the NMJ (Zhang et al., 2007; Wang et al., 2008). Furthermore, studies have suggested that β-catenin in muscle cells (but not in neurons) is involved in the morphology and development of NMJs (Li et al., 2008). In addition, increasing β-catenin expression in muscle leads to increased clustering of ACh receptors (Wu et al., 2012). These data suggest that development of the NMJ requires an intricate balance of β-catenin activity in muscle tissue.

HSA-β-cat^flox(ex3)/+^ mice (i.e., β-catenin gain of function in skeletal muscle cells) are viable at birth, whereas HSA-β-cat^–/–^ mice (i.e., β-catenin deletion of function in skeletal muscle cells) die soon after birth (Li et al., 2008; Wu et al., 2012; Zhao et al., 2014). Furthermore, we have shown that knockdown and disruption of β-catenin depolarizes the RMP in skeletal muscle cells (Zhao et al., 2014). We showed that the RMP in C2C12 cells was refractory to change when we increased β-catenin expression. However, the AP could not be evoked in β-catenin knocked-down or over-expressed C2C12 cells irrespective of the extent of the applied electrical stimulation. These data suggest that deletion (but not an increase) in β-catenin induced depolarization of the RMP in muscle cells. In addition, evoking the AP requires a proper expression of β-catenin in muscle cells. These changes in the electrical properties of muscle cells might contribute to (or result from) abnormalities seen at the NMJ (Jurdana et al., 2009).

### Mechanisms Underlying the Functional Change of Skeletal Muscle Regulated by β-Catenin

Scholars have shown that β-catenin deletion reduces the frequency of miniature endplate potentials (Li et al., 2008; Zhao et al., 2014). We found that the RMP in skeletal muscle cells was regulated by β-catenin expression. In addition, the AP could not be evoked by β-catenin knocked-down or over-expressed C2C12 cells. Changes in β-catenin expression in muscle cells might reduce the excitability of muscle cells (Clausen and Everts, 1991), which might modulate their contractility and fatigability. The mechanism by which β-catenin regulates the electrical properties of skeletal muscle warrants further study.

### Critical Factors in the NKA-Mediated Regulation of the RMP and AP in Muscle Cells

Sodium/potassium adenosine triphosphatase is an integral protein in cell membranes because it maintains the RMP and regulates intracellular and extracellular osmotic balance (Bannett et al., 1984; Brodie et al., 1987). NKA is a heteromer that mainly contains an α-subunit and a glycosylated β-subunit. The α-subunit executes the transport and catalytic activity of NKA (Lingrel and Kuntzweiler, 1994). The β-subunit is also in modulating the affinity of Na^+^ and K^+^ (Eakle et al., 1995). There are four types of α-subunit, and they display tissue-specific expression: the α1 isoform appears in nearly all tissues; the α2 isoform is restricted to smooth muscle, skeletal muscle, and the brain; the α3 subunit is found in neurons and the ovaries; the α4 subunit is found in sperm (Shull et al., 1985, 1986; Sverdlov et al., 1987; Lingrel and Kuntzweiler, 1994).

The skeletal muscles of newborns mainly express the α2 and α1 subunits. Heiny et al. (2010) showed that electrogenic transport by NKA contributes −15 to −20 mV to the RMP of skeletal muscle cells. We found that α2NKA expression decreased if β-catenin expression was deficient, and was increased if β-catenin expression was increased. However, expression of α1NKA and Kv3.4 [both of which can regulate the RMP in skeletal muscle cells (Abbott et al., 2001)] was not altered significantly. We also demonstrated that NKA activity was influenced positively by changes in β-catenin expression in muscle cells.

Sodium/potassium adenosine triphosphatase is central to the excitability, contractility, and fatigability of muscles and nerves (Radzyukevich et al., 2013). In β-catenin knocked-down cells, the AP could not be evoked because cells had been depolarized and were fatigued. Canonical Wnt/β-catenin signaling has been shown to decrease the activity of Na^+^ channels in cardiomyocytes (Wang et al., 2016). In β-catenin over-expressing cells, the generation of AP might be affected not only by α2NKA activity, but also by other ion channels. These data showed a likely mechanism by which β-catenin regulates the RMP and AP and, thus, affects the normal function of skeletal muscle cells.

### β-Catenin Displays a Physical Interaction With α2NKA in Muscle Cells

β-Catenin has two major functions: (i) it acts as a co-transcriptional activator of T-cell factor/lymphocyte enhancer factor-1 and (ii) it regulates expression of target genes (Molenaar et al., 1996; Hecht et al., 2000). β-Catenin also participates in cell adherence (Nelson and Nusse, 2004; Drees et al., 2005). Our study indicated that the effect of β-catenin does not require interaction with α-catenin but, instead, it requires its transactivation domain, which suggests the involvement of transcriptional regulation, but not a cell-adhesion function (Wu et al., 2015). Our study showed that the mRNA expression of α2NKA changed in accordance with altered functional expression of β-catenin. In addition, studies have shown that endogenous NKA current is enhanced by β-catenin over-expression (Sopjani et al., 2010). Thus, β-catenin is likely to regulate α2NKA by its co-transcriptional function.

Furthermore, our results suggested that β-catenin and α2NKA exerted a physical interaction with C2C12 cells. We also revealed that NKA activity and α2NKA content in the cell membrane were modulated positively by β-catenin.

## Conclusion

We provided further evidence that β-catenin is crucial for maintaining the RMP and evoking the AP in skeletal muscle cells. Skeletal muscle cells lacking β-catenin expression and with high expression of β-catenin showed decreased expression and increased expression of α2NKA, respectively. Hence, β-catenin appears to regulate the electrical properties and affects the normal functioning of skeletal muscle cells.

We showed that β-catenin could interact physically with α2NKA (but not with α1NKA) in muscle cells. β-Catenin could regulate the enzymatic activity and content of α2NKA in cell membranes. These results suggested that β-catenin (at least in part) regulates the RMP and AP in muscle cells, and does so by regulating α2NKA.

## Data Availability

The raw data supporting the conclusions of this manuscript will be made available by the authors, without undue reservation, to any qualified researcher.

## Author Contributions

FG, CZ, KJ, YY, and JS conceptualized and designed the study. CZ, YY, YZ, JS, LJ, GS, LX, and WZ acquired and analyzed the data. CZ, SM, and PJ drafted the text and prepared the figures. All authors approved the final version of the manuscript to be published.

## Conflict of Interest Statement

The authors declare that the research was conducted in the absence of any commercial or financial relationships that could be construed as a potential conflict of interest.
