# Supplementary material for: β-Catenin Controls the Electrophysiologic Properties of Skeletal Muscle Cells by Regulating the α2 Isoform of Na+/K+-ATPase
Source: Front Neurosci. 2019 Aug 7;13:831. doi: 10.3389/fnins.2019.00831 (PMC6693565; doi:10.3389/fnins.2019.00831)
Supplement: Supplementary file 1 [file Table_1.DOCX]

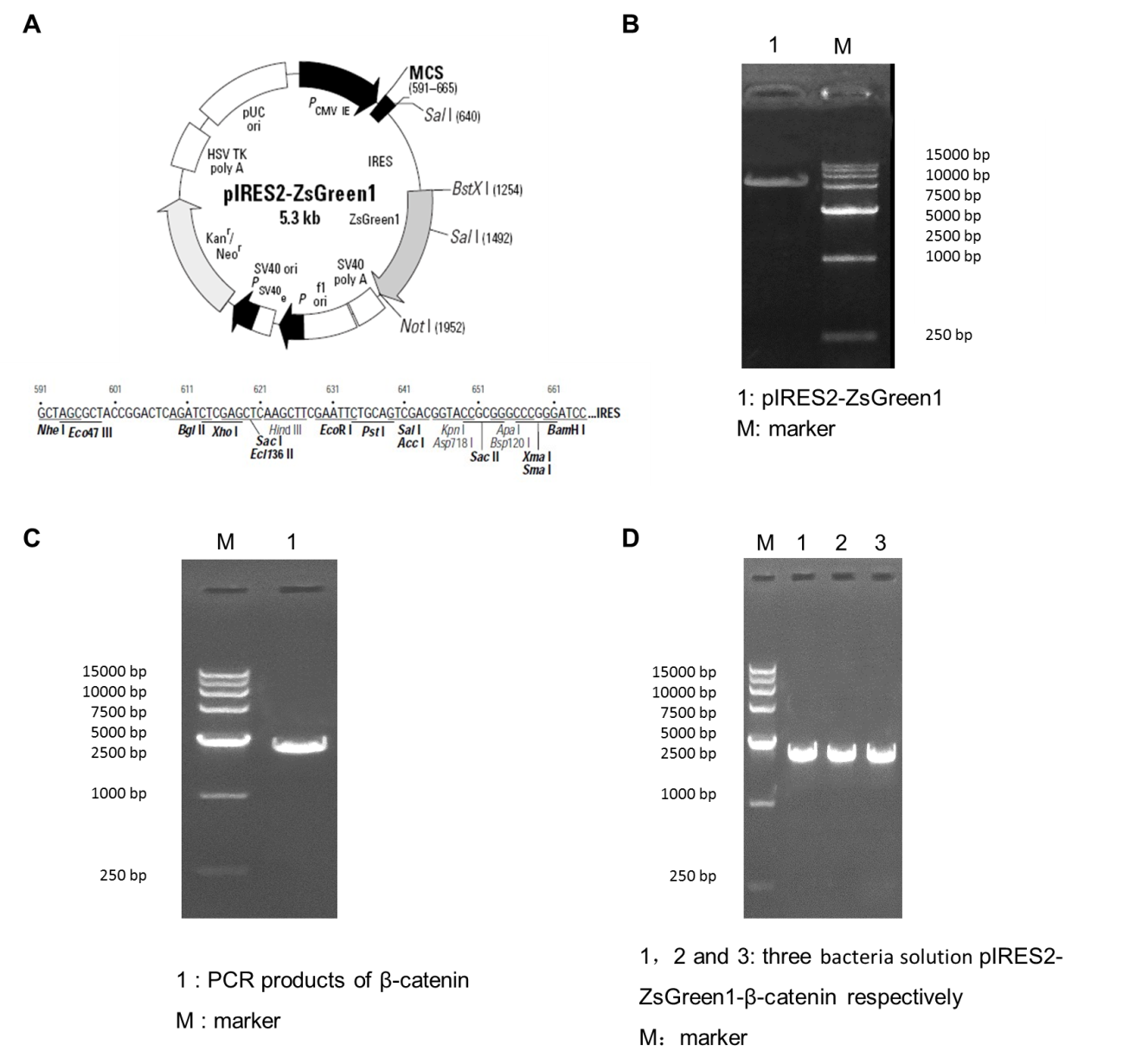


**Supplementary Figure 1.** Construction of the overexpression plasmid of β-catenin**.** (A) The linearized pIRES2-ZsGreen1 construct. The vector was digested by the restriction endonucleases XhoI and EcoRI. (B) The digested pIRES2-ZsGreen1 was subjected to 1% *w/v* agarose gel electrophoresis, and then tapped and recovered. (C) The primers were used as templates, and each was adopted to obtain β-catenin as the foreign-insert DNAs for PCR cloning. PCR products of β-catenin were ~2500 bp, which was consistent with the target β-catenin gene of 2346 bp. (D) Identification of cultured bacteria by PCR. The PCR products of β-catenin were cloned into the prokaryotic expression vector pIRES2-ZsGreen1. Then, the vector was transformed into *Escherichia coli* DH5a cells to construct a prokaryotic expression system.

**
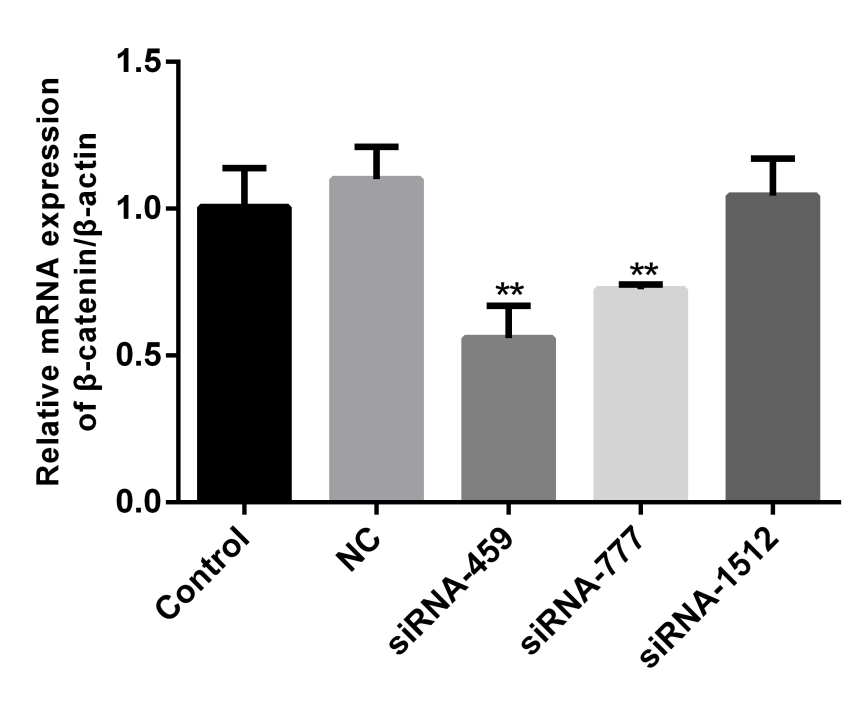
**

**Supplementary Figure 2.** Absolute transcription levels of β-catenin in siRNA-transfected C2C12 cells from more than three experiments, with values obtained from the control normalized as 1. Compared with control cells, β-catenin mRNA in β-catenin siRNA459 and siRNA777 groups was decreased significantly (*P* < 0.01) but this effect was not seen in β-catenin siRNA1512 cells (*P* > 0.05). ***P* < 0.01. Error bars represents SEM.


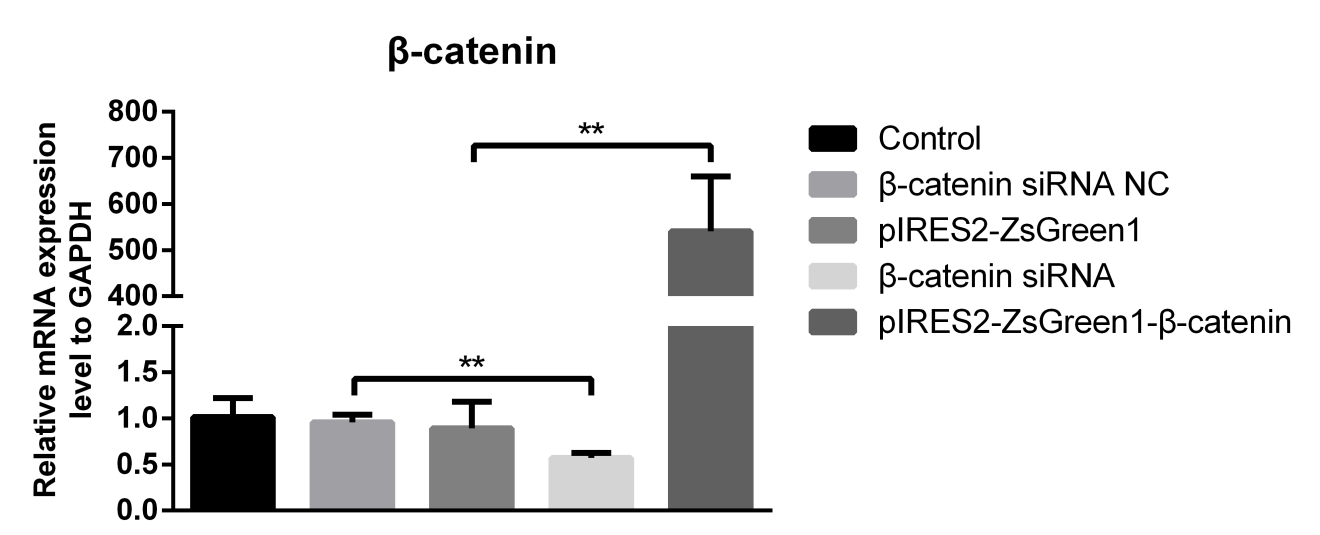


**Supplementary Figure 3.** Absolute transcription levels of β-catenin in five transfection groups from more than three experiments, with values obtained from the control normalized as 1. Compared with β-catenin siRNA normal control cells, expression of β-catenin mRNA in β-catenin siRNA cells was reduced significantly (t-test, *P* = 0.0027). Compared with pIRES2-ZsGreen1cells, expression of β-catenin mRNA in pIRES2-ZsGreen1-β-catenin cells was increased significantly (t-test, *P* = 0.0014). ***P* < 0.01. Error bars represents SEM.
